# Supplementary material for: Floating photovoltaic systems: photovoltaic cable submersion testing and potential impacts
Source: Open Res Eur. 2024 Sep 16;3:61. Originally published 2023 Apr 24. [Version 2] doi: 10.12688/openreseurope.15122.2 (PMC11459181; doi:10.12688/openreseurope.15122.2)
Supplement: Peer review history

# Reviewer report: Reviewer response for version 2

**DOI:** 10.21956/openreseurope.20030.r44368

**Published:** 2024-10-09

**Authors:** Shiva Gorjian

**Affiliations:** 1 Biosystems Engineering Department, Faculty of Agriculture, Tarbiat Modares University (TMU), Tehran, India

The authors have properly addressed the comments and the revised version is satisfactory.

Is the study design appropriate and does the work have academic merit?

Partly

Is the work clearly and accurately presented and does it cite the current literature?

Partly

If applicable, is the statistical analysis and its interpretation appropriate?

Not applicable

Are all the source data underlying the results available to ensure full reproducibility?

Partly

Are the conclusions drawn adequately supported by the results?

Partly

Are sufficient details of methods and analysis provided to allow replication by others?

Partly

Reviewer Expertise:

Renewable Energies; Solar Energy Technologies

I confirm that I have read this submission and believe that I have an appropriate level of expertise to confirm that it is of an acceptable scientific standard.

# Reviewer report: Reviewer response for version 2

**DOI:** 10.21956/openreseurope.20030.r44367

**Published:** 2024-10-07

**Authors:** Sharon Hilarydoss

**Affiliations:** 1 Department of Mechanical Engineering, Indian Institute of Petroleum and Energy (IIPE), Visakhapatnam, Andhra Pradesh, India

My comments have been addressed

Is the study design appropriate and does the work have academic merit?

Yes

Is the work clearly and accurately presented and does it cite the current literature?

Yes

If applicable, is the statistical analysis and its interpretation appropriate?

I cannot comment. A qualified statistician is required.

Are all the source data underlying the results available to ensure full reproducibility?

Yes

Are the conclusions drawn adequately supported by the results?

Partly

Are sufficient details of methods and analysis provided to allow replication by others?

Partly

Reviewer Expertise:

solar energy, desalination, thermodynamic analysis,

I confirm that I have read this submission and believe that I have an appropriate level of expertise to confirm that it is of an acceptable scientific standard.

# Reviewer report: Reviewer response for version 2

**DOI:** 10.21956/openreseurope.20030.r44376

**Published:** 2024-09-20

**Authors:** Giuseppe Marco Tina

**Affiliations:** 1 University of Catania, Catania, Italy

Overall, the paper provides valuable insights into the behavior of photovoltaic (PV) cables submerged in two different water types. However, the limited scope of the experiments and the need for further analysis warrant careful consideration of the findings.

 Key observations and suggestions:

 references:

 references and  standards for suitable cable in aquatic and marine environments should be cited. such as https://doi.org/10.4043/2428-A-MS

 Cable Submersion:

 The solar  gables are used to connect the PV modules and also to connect the string to the inverter that is supposed to be installed on the same floating structure. So it is not common to have such cables always submerged in floating PV plants. It could be happen in submerged PV plants such as the ones described in reference 7. In case of cable always submerged adequate types of cable should be used according to the standards  especially if we are in marine environment.

 While submerged cables are less common in floating PV plants, the authors' focus on this aspect is valuable.

 The use of appropriate cable types, especially in marine environments, is critical for long-term performance.

 Experimental Conditions: The experiments provide a solid foundation for understanding cable behavior in different water types. However, expanding the scope to include a wider range of environmental conditions, such as varying temperatures and salinity levels, could enhance the generalizability of the results.

 Insulation Resistance: The lack of significant insulation resistance reduction for most cables is encouraging (see Fig. 7). However, the singularity observed in cable 2 in saltwater during the eighth week warrants further investigation. It's possible that a material defect or a temporary environmental fluctuation contributed to this anomaly.  A statistical approach has to be used to present the results.

 Container Degradation: To isolate the effects of the cables on the container material, it would be beneficial to include a control group of containers without cables. This would help determine if the container material itself is susceptible to degradation in the experimental conditions.

 Cable Shape and Stress: The coiled shape of the cables in the experiments might not accurately reflect their real-world configuration. Considering the potential impact of cable tension and stress, especially at low temperatures, could provide additional insights into insulation performance.

 Current Warming: While current flow can indeed warm the cables, the extent to which this mitigates the effects of low temperatures depends on factors such as cable thickness, current intensity, and the specific environmental conditions. Further analysis or simulations could quantify this effect.

 In conclusion, the paper's findings contribute to our understanding of PV cable behavior in submerged environments. However, additional experiments and a more comprehensive analysis of factors such as environmental conditions, cable shape, and current warming are necessary to draw definitive conclusions about the long-term performance of these cables.

Is the study design appropriate and does the work have academic merit?

Partly

Is the work clearly and accurately presented and does it cite the current literature?

Partly

If applicable, is the statistical analysis and its interpretation appropriate?

I cannot comment. A qualified statistician is required.

Are all the source data underlying the results available to ensure full reproducibility?

Yes

Are the conclusions drawn adequately supported by the results?

Partly

Are sufficient details of methods and analysis provided to allow replication by others?

Partly

Reviewer Expertise:

photovoltaic systems, power system analysis

I confirm that I have read this submission and believe that I have an appropriate level of expertise to confirm that it is of an acceptable scientific standard, however I have significant reservations, as outlined above.

# Reviewer report: Reviewer response for version 1

**DOI:** 10.21956/openreseurope.16359.r31511

**Published:** 2023-06-16

**Authors:** Shiva Gorjian

**Affiliations:** 1 Biosystems Engineering Department, Faculty of Agriculture, Tarbiat Modares University (TMU), Tehran, India

The authors have investigated “Floating photovoltaic systems: photovoltaic cable submersion testing and potential impacts”. The following comments should be addressed:

1. In Figure 2, it is better to add data for the years 2021-2023.
2. Based on which criteria the authors have chosen the salinity level? Since FPVs can be installed on seawater, lakes, artificial water bodies, etc.
3. Has the resistance time been considered as a parameter to evaluate the degradation level of the PV cables? If yes, how can it be generalized to real conditions?
4. According to the results, can water temperature be considered an effective parameter in combination with water salinity?
5. The results and discussion section should be improved.

Is the study design appropriate and does the work have academic merit?

Partly

Is the work clearly and accurately presented and does it cite the current literature?

Partly

If applicable, is the statistical analysis and its interpretation appropriate?

Not applicable

Are all the source data underlying the results available to ensure full reproducibility?

Partly

Are the conclusions drawn adequately supported by the results?

Partly

Are sufficient details of methods and analysis provided to allow replication by others?

Partly

Reviewer Expertise:

Renewable Energies; Solar Energy Technologies

I confirm that I have read this submission and believe that I have an appropriate level of expertise to confirm that it is of an acceptable scientific standard, however I have significant reservations, as outlined above.

# Response

**Published:** 2024-09-03

**Authors:** Luis FIALHO

The authors appreciate the reviewer's comments. All comments have been addressed in this version of the document, and we believe they resolve the issues raised. The authors did not collect installed capacity data beyond the final date shown in the graph already included in the article. Regarding comment 3: The authors would like to ask the reviewer to provide a bit more detail about what he intends to make clearer with it. The tests were conducted in a near-real environment, where conditions such as temperature, humidity, etc., vary over time, making it impossible to define the resistance time with precision. In the authors' opinion, temperature and salinity are influential parameters in the degradation of submerged cables, requiring further testing to obtain a more representative sample.

# Reviewer report: Reviewer response for version 1

**DOI:** 10.21956/openreseurope.16359.r31515

**Published:** 2023-05-17

**Authors:** Sharon Hilarydoss

**Affiliations:** 1 Department of Mechanical Engineering, Indian Institute of Petroleum and Energy (IIPE), Visakhapatnam, Andhra Pradesh, India

- Follow uniform decimal format throughout the manuscript including figures.
- Indicate components by markings in Fig 4, 5, 6 , 8.
- English language usage must be improved throughout the manuscript.
- Check the spelling of the sub heading "Water physic-chemical characterization".
- Superscripts are not properly indicated, check them throughout the manuscript
- Conclusion section must be revised by shortening it and including only important findings. Move discussion portion from conclusion. In future work section below conclusion section.

Is the study design appropriate and does the work have academic merit?

Yes

Is the work clearly and accurately presented and does it cite the current literature?

Yes

If applicable, is the statistical analysis and its interpretation appropriate?

I cannot comment. A qualified statistician is required.

Are all the source data underlying the results available to ensure full reproducibility?

Yes

Are the conclusions drawn adequately supported by the results?

Partly

Are sufficient details of methods and analysis provided to allow replication by others?

Partly

Reviewer Expertise:

solar energy, desalination, thermodynamic analysis,

I confirm that I have read this submission and believe that I have an appropriate level of expertise to confirm that it is of an acceptable scientific standard, however I have significant reservations, as outlined above.

# Response

**Published:** 2024-09-03

**Authors:** Luis FIALHO

The authors appreciate the reviewer's comments. All comments have been addressed in this version of the document, and we believe they resolve the issues raised.
